# Long intergenic non-protein coding RNA 467 inhibition elevates microRNA-27b-3p to repress malignant behaviors of gastric cancer cells via reducing STAT3

**DOI:** 10.1038/s41420-022-00875-z

**Published:** 2022-03-05

**Authors:** Mingdian Lu, Dong Liu, Yongxiang Li

**Affiliations:** Department of Gastrointestinal Surgery and General Surgery, First Affiliated Hospital of Anhui Medical University, Hefei, 230022 Anhui People’s Republic of China

**Keywords:** Cancer, Diseases

## Abstract

Emerging evidence indicated that long noncoding RNAs (lncRNAs) and microRNAs (miRNAs) exert critical effects on tumorigenesis of multiple malignancies, including gastric cancer (GC). We aim to explore the effects of long intergenic non-protein coding RNA 467 (LINC00467) and miR-27b-3p on GC. GC cells were initially cultured. LINC00467, miR-27b-3p, and signal transducer and activator of transcription 3 (STAT3) expression in GC were detected. The altered LINC00467 and/or miR-27b-3p were transfected into screened cells. Then, the biological activities of GC cells and the tumor growth in vivo were examined. The binding relationships among LINC00467, miR-27b-3p, and STAT3 were confirmed. It was indicated that LINC00467 was increased while miR-27b-3p was decreased in GC tissues and cells. Inhibition of LINC00467 hindered GC cell malignancy and blocked tumor development by upregulating miR-27b-3p. LINC00467 sponged miR-27b-3p and STAT3 was targeted by miR-27b-3p. It was discovered that LINC00467 reduction upregulates miR-27b-3p to repress malignant GC cell growth via inhibiting STAT3. This research may deepen the insight of molecular mechanisms on GC.

## Introduction

Gastric cancer (GC) is the 5th most prevalent cancer worldwide and the 3rd major inducer of cancer-related deaths, and may arise in the cardia or non-cardia [1]. In most countries, the ratio of mortality and incidence of GC cases is more than 0.8, which is partly attributed to the late detection caused by the deficiency of particular symptoms and the limitation of therapeutic options for advanced disease [2]. With the elevation of incidence and mortality rates of GC worldwide, GC is predicted to be one of the top 15 leading causes of deaths among all diseases in 2020 and 2030 [3]. The outcome of GC is poor as most patients are diagnosed with disseminated diseases, and this is possibly caused by the lack of non-invasive and early diagnostic tools [4]. Although some treatment methods have been demonstrated to improve the outcome of GC patients, the prognosis is still poor, and the median survival rate is about 1 year [5]. Therefore, novel biomarkers need to be explored for the improvement of GC treatment.

Long noncoding RNAs (lncRNAs) are a set of ncRNAs with over 200 nt and a deficiency of protein-coding ability [6]. Some particular lncRNAs, such as lncRNA H19 and lncRNA HOTAIR [7, 8] were implicated in the GC progression. LncRNA long intergenic non-protein coding RNA 00467 (LINC00467) is an oncogenic lncRNA in multiple cancers, such as cervical cancer [9] and lung adenocarcinoma [10]. Nevertheless, the role of LINC00467 has not been identified in GC. Moreover, lncRNAs can serve as sponges to absorb miRNAs and abolish the degradation of target genes induced by miRNAs [11]. The bioinformatics websites predicted binding sites between LINC00467 and miR-27b-3p. As reported, miR-27b-3p was demonstrated to be a tumor repressor in GC [12, 13], while the combinative effect of LINC00467 and miR-27b-3p on the disease has not been unveiled. Furthermore, the current study disclosed that miR-27b-3p had a targeting relation with signal transducer and activator of transcription 3 (STAT3). The mechanism of STAT3 in GC has been widely studied [14, 15], and it was unraveled that the c-Src/STAT3 signaling pathway served as a active participant in miR-27b-3p-mediated proliferative activities of GC cells [12].

As stated above, LINC00467, miR-27b-3p, and STAT3 exerted crucial influences in cancer progression, especially in CG. Our study further discovered the binding relation among LINC00467, miR-27b-3p, and STAT3 through the bioinformatic database. In light of this, we hypothesized that LINC00467 might regulate the biological functions of GC cells through modulating the miR-27b-3p/STAT3 axis, thus providing novel therapeutic candidates for GC treatment.

## Results

### LINC00467 and STAT3 levels are elevated while miR-27b-3p is depleted in GC tissues

**Fig. 1:**
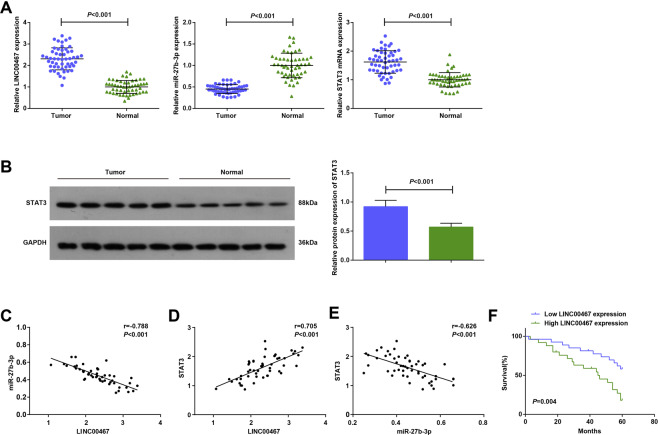
LINC00467 and STAT3 are elevated while miR-27b-3p is depleted in GC tissues. (**A**) expression of LINC00467, miR-27b-3p, and STAT3 in GC tissues and adjacent normal tissues. (**B**) Protein band and protein expression of STAT3 in GC tissues and adjacent normal tissues. (**C**) Relation between levels of LINC00467 and miR-27b-3p in GC patients was analyzed by Pearson test. (**D**) Relation between levels of LINC00467 and STAT3 in GC patients was analyzed by Pearson test. (**E**) Relation between levels of miR-27b-3p and STAT3 in GC patients was analyzed by Pearson test. (**F**) Survival prognosis of GC patients was analyzed by Kaplan–Meier; *n* = 52; * *P* < 0.05 vs the adjacent normal tissues; the measurement data were expressed as mean ± standard deviation.

Expression of LINC00467, miR-27b-3p, and STAT3 in tissues was evaluated and it indicated that (Fig. 1A, B) the levels of LINC00467 and STAT3 were higher, while miR-27b-3p expression was decreased in GC tissues.

As reflected by the Pearson test (Fig. 1C–E), LINC00467 expression was negatively correlated with miR-27b-3p expression, while the miR-27b-3p level was negatively related with STAT3 expression, and LINC00467 expression was positively associated with STAT3 expression (all *P* < 0.05).

Subsequently, the correlation between the relative expression of LINC00467 and the clinicopathological characteristics of GC patients was unraveled. The patients were classified into low expression group (*n* = 26) and high expression group (*n* = 26) concerning the median value of LINC00467 level. The results uncovered that high LINC00467 level was implicated in pathological stage, lymph node metastasis, and tumor differentiation; however, LINC00467 expression was not associated with tumor location or patient gender or age (Supplementary Table 2).

Kaplan–Meier was employed to examine the effects of LINC00467 on the survival prognosis of GC patients. It turned out that the difference in survival time between the high expression group and the low expression group was statistically significant (*P* < 0.05). Specifically, the high-expressed LINC00467 indicated the worsened prognosis of GC patients (Fig. 1F).

### LINC00467 and STAT3 are increased while miR-27b-3p is decreased in GC cells

**Fig. 2:**
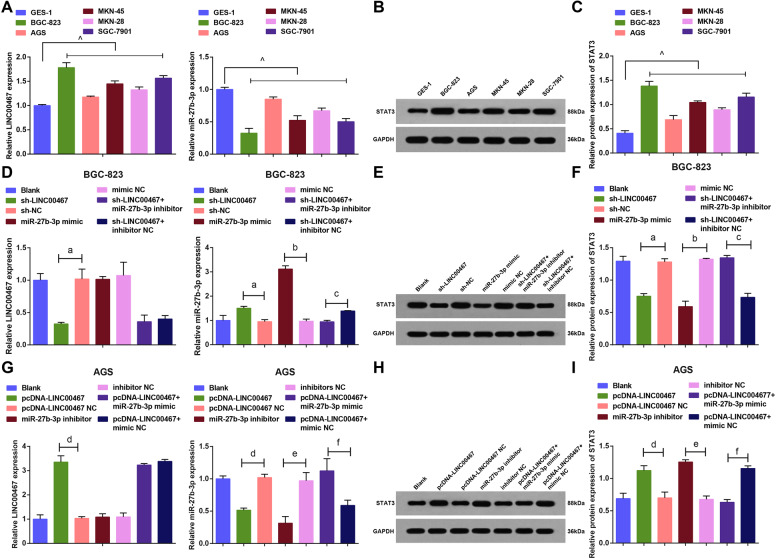
LINC00467 and STAT3 are increased while miR-27b-3p is decreased in GC cells. (**A**) Expression of LINC00467, and miR-27b-3p in GES-1 cells and GC cell lines. (**B**) Protein band of STAT3 in GES-1 cells and GC cell lines. (**C**) Protein expression of STAT3 in GES-1 cells and GC cell lines. (**D**) Expression of LINC00467, and miR-27b-3p in BGC-823 cells of each group. **E** Protein band of STAT3 in BGC-823 cells of each group. **F** Protein expression of STAT3 in BGC-823 cells of each group. **G** Expression of LINC00467, and miR-27b-3p in AGS cells of each group. **H** Protein band of STAT3 in AGS cells of each group. **I** Protein expression of STAT3 in AGS cells of each group; ^ *P* < 0.05 vs GES-1 cells; a *P* < 0.05 *vs* the sh-NC group, b *P* < 0.05 *vs* the mimic NC group, c *P* < 0.05 vs the sh-LINC00467 + inhibitor NC group, d *P* < 0.05 vs the pcDNA-LINC00467 NC group, e *P* < 0.05 vs the inhibitor NC group, f *P* < 0.05 vs the pcDNA-LINC00467 + mimic NC group; *N* = 3; the measurement data were expressed as mean ± standard deviation.

LINC00467, miR-27b-3p, and STAT3 expression in GES-1 cells and GC cell lines were measured and the results revealed that (Fig. 2A–C) relative to GES-1 cells, GC cell lines had higher levels of LINC00467 and STAT3, and a lower level of miR-27b-3p. BGC-823 cells exhibited the maximum, while AGS cells displayed the minimum expression difference of LINC00467, miR-27b-3p, and STAT3 from GES-1 cells. Thus, BGC-823 and AGS were selected for subsequent experiments.

In BGC-823 cells (Fig. 2D–F), there exhibited no marked difference in LINC00467, miR-27b-3p, and STAT3 levels among the blank, sh-NC, and mimic NC groups. After treated with silenced LINC00467 and miR-27b-3p mimic/inhibitor, silenced LINC00467 plasmid inhibited the expression of LINC00467 and STAT3 but upregulated miR-27b-3p; miR-27b-3p mimic upregulated miR-27b-3p while downregulated STAT3.

In AGS cells (Fig. 2G–I), LINC00467 and STAT3 levels were increased while miR-27b-3p displayed a low level in the pcDNA-LINC00467 group; miR-27b-3p was downregulated but STAT3 was upregulated in cells that subjected to the miR-27b-3p inhibitor.

### LINC00467 silencing or miR-27b-3p amplification restrains malignant behaviors of GC cells

**Fig. 3:**
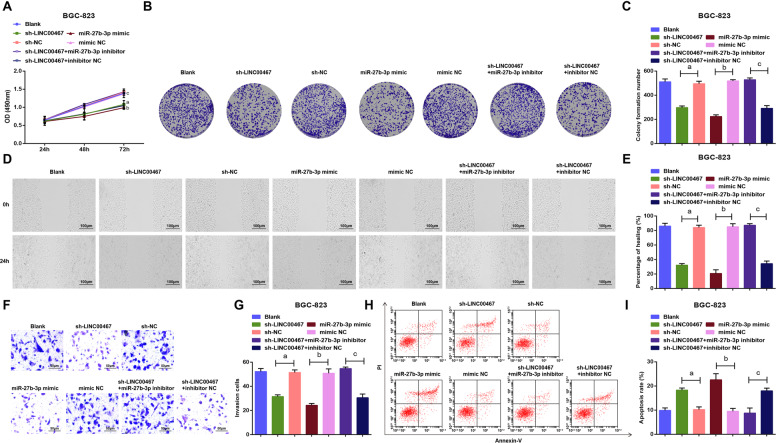
Knockdown of LINC00467 or elevation of miR-27b-3p suppresses malignant behaviors of GC cells. **A** BGC-823 cell growth was measured by MTS colorimetry. **B** Colony formation ability of BGC-823 cells was detected by colony formation assay. **C** Number of colonies of BGC-823 cells. **D** Migration ability of BGC-823 cells was measured by scratch test. **E** Comparison of healing rate of BGC-823 cells among the groups. **F** Invasion ability of BGC-823 cells was measured by Transwell assay. **G** Comparison of invasive ability of BGC-823 cells among the groups. **H** Apoptosis rate of BGC-823 cells in each group. **I** Apoptosis of BGC-823 cells was gauged by flow cytometry; a *P* < 0.05 vs the sh-NC group, b *P* < 0.05 vs the mimic NC group, c *P* < 0.05 vs the sh-LINC00467 + inhibitor NC group; *N* = 3; the measurement data were expressed as mean ± standard deviation.

Cell activities were evaluated and the outcomes implied that in BGC-823 cells (Fig. 3A–I), the cell viability, healing rate, and the number of invasive cells were all reduced, while the apoptosis rate was enhanced in the sh-LINC00467 or miR-27b-3p mimic groups; miR-27b-3p inhibitor inverted the effect of silenced LINC00467 on cell viability, healing rate, number of invasive cells and apoptosis rate.

### Upregulation of LINC00467 or inhibition of miR-27b-3p promotes malignant phenotypes of GC cells

**Fig. 4:**
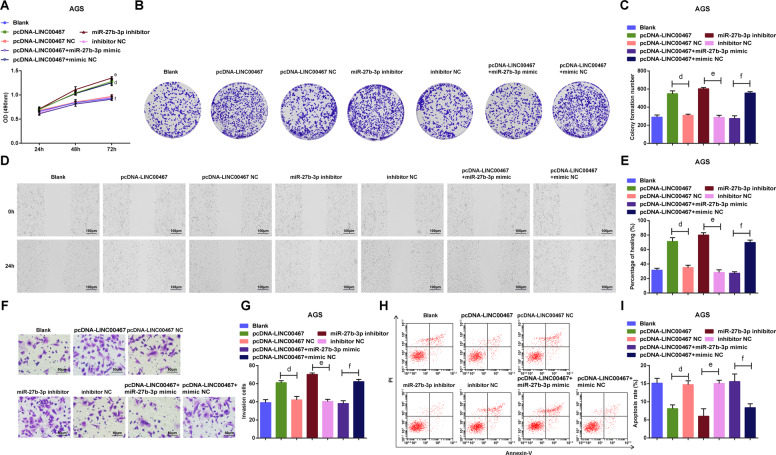
Upregulation of LINC00467 or inhibition of miR-27b-3p promotes malignant phenotypes of GC cells. **A** AGS cell growth was measured by MTS colorimetry. **B** Colony formation ability of AGS cells was detected by colony formation assay. **C** number of colonies of AGS cells. **D** Migration ability of AGS cells was measured by scratch test. **E** comparison of healing rate of AGS cells among the groups. **F** Invasion ability of AGS cells was measured by Transwell assay. **G** comparison of invasive ability of AGS cells among the groups. **H** Apoptosis rate of AGS cells in each group. **I** Apoptosis of AGS cells was gauged by flow cytometry; d *P* < 0.05 vs the pcDNA-LINC00467 NC group, e *P* < 0.05 vs the inhibitor NC group, f *P* < 0.05 vs the pcDNA-LINC00467 + mimic NC group; *N* = 3; the measurement data were expressed as mean ± standard deviation.

After the examination of cell biological activities, we discovered that in AGS cells (Fig. 4A–I), the cell viability, healing rate and the number of invasive cells were all accelerated; while the apoptosis rate was constrained in the pcDNA-LINC00467 or miR-27b-3p inhibitor groups; transfection of miR-27b-3p mimic reversed the impact of overexpressed LINC00467 on cell viability, healing rate, number of invasive cells and apoptosis rate.

### Knockdown of LINC00467 or elevation of miR-27b-3p decelerates GC tumor growth in vivo

**Fig. 5:**
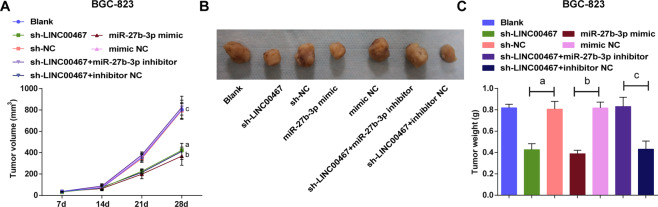
Knockdown of LINC00467 or elevation of miR-27b-3p decelerates GC tumor growth in vivo. **A** Growth curve of BGC-823 xenografts. **B** Representative images of BGC-823 xenografts. **C** Weight of BGC-823 xenografts in each group; a *P* < 0.05 vs the sh-NC group, b *P* < 0.05 *vs* the mimic NC group, c *P* < 0.05 vs the sh-LINC00467 + inhibitor NC group; *n* = 6; the measurement data were expressed as mean ± standard deviation.

The tumor growth in vivo was observed and results in BGC-823 xenografts indicated that (Fig. 5A–C) tumor volume and weight were hindered in the sh-LINC00467 or miR-27b-3p mimic groups versus their NC groups; miR-27b-3p depletion abrogated the effect of inhibited LINC00467.

### Upregulation of LINC00467 or miR-27b-3p decrement accelerates GC tumor growth in vivo

**Fig. 6:**
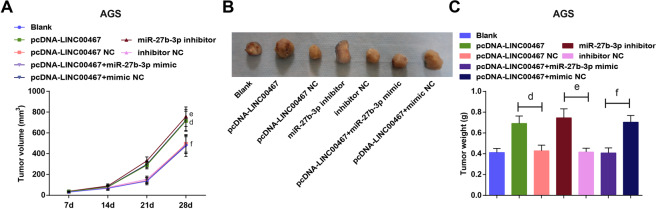
Upregulation of LINC00467 or inhibition of miR-27b-3p accelerates GC tumor growth in vivo. **A** growth curve of AGS xenografts. **B** Representative images of AGS xenografts. **C** Weight of AGS xenografts in each group; d *P* < 0.05 vs the pcDNA-LINC00467 NC group, e *P* < 0.05 vs the inhibitor NC group, f *P* < 0.05 vs the pcDNA-LINC00467 + mimic NC group; *n* = 6; the measurement data were expressed as mean ± standard deviation.

Results of observation of xenografts in nude mice mirrored that (Fig. 6A–C) tumor growth was dampened in the pcDNA-LINC00467 or miR-27b-3p inhibitor groups versus their NC groups; miR-27b-3p mimic inverted the impacts of overexpressed LINC00467 in elevating tumor volume and weight.

### LINC00467 sponges miR-27b-3p to regulate STAT3

**Fig. 7:**
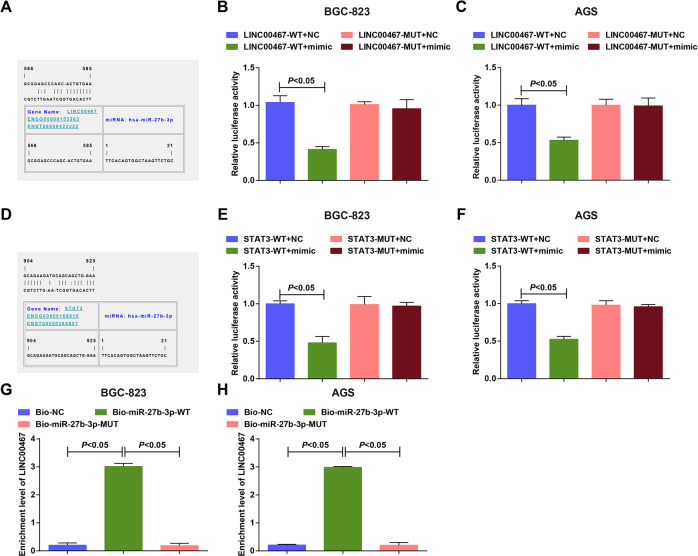
LINC00467 serves as a ceRNA to sponge miR-27b-3p, thereby regulating STAT3. **A** Binding sites of LINC00467 and miR-27b-3p were predicted by bioinformatics website. **B** Regulatory relation between LINC00467 and miR-27b-3p in BGC-823 cells was confirmed by dual-luciferase reporter gene assay. **C** Regulatory relation between LINC00467 and miR-27b-3p in AGS cells was confirmed by dual-luciferase reporter gene assay. **D** Target relation between miR-27b-3p and STAT3 was predicted by bioinformatics website. **E** Target relation between miR-27b-3p and STAT3 in BGC-823 cells was confirmed by dual-luciferase reporter gene assay. **F** Target relation between miR-27b-3p and STAT3 in AGS cells was confirmed by dual-luciferase reporter gene assay. **G** Binding relation between LINC00467 and miR-27b-3p in BGC-823 cells was confirmed by RNA pull-down assay. **H** Binding relation between LINC00467 and miR-27b-3p in AGS cells was confirmed by RNA pull-down assay; *N* = 3; the measurement data were expressed as mean ± standard deviation.

As predicted by the bioinformatics website (https://cm.jefferson.edu/rna22/Precomputed/), a particular binding region between the sequences of LINC00467 and miR-27b-3p were confirmed (Fig. 7A). It was further validated that (Fig. 7B, C), miR-27b-3p mimic inhibited luciferase activities of LINC00467-WT vectors but not the mutant vectors, revealing a binding relation between LINC00467 and miR-27b-3p.

It was further predicted by bioinformatics software that miR-27b-3p and STAT3 had a targeting relation (Fig. 7D). As further confirmed (Fig. 7E, F), luciferase activity was markedly lower in STAT3-WT vectors transfected cells that were co-transfected with the miR-27b-3p mimic. However, no inhibition of luciferase activity was observed for cells that were transfected with STAT3-MUT vectors, uncovering that STAT3 was the target of miR-27b-3p.

The outcomes of RNA pull-down assay (Fig. 7G, H) implied that in BGC-823 and AGS cells, the bio-miR-27b-3p-WT group had a higher expression of LINC00467 (*P* < 0.05). These data indicated that bio-miR-27b-3p-WT promoted the enrichment of LINC00467, suggesting that LINC00467 may bind to miR-27b-3p and repress the free degree of miR-27b-3p.

## Discussion

GC incidence and mortality have been reduced in the recent decades, while the global burden of GC is estimated to enhance in the future years due to the demographic effect of growth and aging of the population in the world [16]. Noncoding regions account for over 90% of the human genome, and exerted vital influences in the modulation of physiological function. As a representative of noncoding regions, approximately 18% of lncRNAs are believed to be correlated with human cancers [17]. This study aims to unravel the mechanism of the lncRNA LINC00467/miR-27b-3p/STAT3 axis in GC, and we affirmed that LINC00467 knockdown upregulated miR-27b-3p to suppress malignant phenotypes of GC cells through reducing STAT3.

LINC00467, miR-27b-3p, and STAT3 expression in GC tissues and cells were determined. Outcomes revealed that LINC00467 and STAT3 were upregulated while miR-27-3p was downregulated in GC, separately in comparison to that in adjacent normal tissues and GES-1 cells. Consistently, it was found that LINC00467 is highly expressed in cervical cancer [9], and Wang et al. Have noted that LINC00467 is increased in lung tumor tissues versus the normal tissues [10]. It has been identified that miR-27b-3p is deficient in GC cell lines and tissues in relation to the normal group [13], and this downregulation of miR-27b-3p in GC has been verified by Tao et al. as well [12]. Furthermore, Zhang et al. have clarified that STAT3 level in patients with advanced GC, early GC and gastric precancerous lesions is higher than in those with normal gastric mucosa [14]. Moreover, the immunohistochemical expression of STAT3 in tumor-infiltrated areas is nearly three times higher than that in tissues selected from adjacent non-tumor regions [15]. Furthermore, we have also clarified that LINC00467 could absorb miR-27b-3p, thereby negatively regulating its expression, and the target relation between miR-27b-3p and STAT3 was validated. However, both relationships have not been uncovered before.

Through the gain- and loss-of-function assays, the research further manifested the roles of altered LINC00467 and miR-27b-3p in the malignant activities of GC cells. The GC cell lines were accordingly treated with silenced or overexpressed LINC467, and/or miR-27b-3p mimic or inhibitor. One of the outcomes in our cellular experiments suggested that the reduction of LINC00467 and transfection of miR-27b-3p mimic contribute to suppressing the growth of GC cells in vitro. Similar to this finding, it was recently affirmed that LINC00467 silencing constrains the malignant biological behaviors of colorectal cancer cells in vitro [18], and Jiang et al. have demonstrated that reduced LINC00467 represses proliferation and metastasis of hepatocellular carcinoma (HCC) cells [19]. Additionally, the upregulation of miR-27b-3p has been validated to block malignant episodes of GC cells [13]. Another finding in this research reflected that LINC00467 silencing promoted miR-27b-3p expression to accelerate apoptosis of GC cells. By this result, it has been unveiled that LINC00467 knockdown induces the apoptosis of HCC cells [19], and Chang et al. have unearthed that the deletion of LINC00467 facilitates the apoptosis of lung adenocarcinoma cells [20]. Moreover, a publication has implied that miR-27b-3p serves as a tumor repressor in lung cancer and promotes apoptosis of lung cancer cells [21]. The impacts of LINC00467 and miR-27b-3p on GC tumor development in vivo were validated by subcutaneous tumorigenesis, and it came out that the repressed LINC00467 and elevated miR-27b-3p decelerated GC tumor growth. Consistently, Ding et al. have figured out that the downregulated LINC00467 hinders the growth of lung adenocarcinoma in vivo [11], and it has been unraveled that miR-27b-3p augmentation is capable of suppressing xenograft tumor growth of GC cells [12].

In summary, it was disclosed that the inhibition of LINC00467 elevated miR-27b-3p to constrain malignant progression of GC cells by declining STAT3. This research may shed light on the functional mechanisms of LINC00467 and miR-27b-3p on GC progression, while the elucidation requires further research.

## Materials and methods

### Ethics statement

All patients have signed the written informed consents before this research. Animal experiments were supervised by the Institutional Animal Care and Use Committee of First Affiliated Hospital of Anhui Medical University.

### Study subjects

Fifty-two GC tissues from patients diagnosed as GC and accepted treatment in First Affiliated Hospital of Anhui Medical University together with the adjacent normal tissues (3–5 cm from the tumor edge) were preserved in liquid nitrogen. Among the 52 patients (aged 31–75 years, mean age of 58.2 years), there were 31 males and 21 females. Patients that had accepted radio- or chemotherapy were excluded. The pathological tissues were diagnosed by gastroscopic biopsies. According to Cancer Staging Manual 6th Edition by the American Joint Committee on Cancer, the cases were classified based on tumor, node, and metastasis (TNM) stage: I stage (10), II stage (16), III stage (15), and IV stage (11).

### Cell culture

Gastric mucosal epithelial cell line GES-1 was offered by Obio Technology Corp., Ltd. (Shanghai, China), and the GC cell lines BGC-823, AGS, MKN-45, MKN-28, and SGC-7901 were all obtained from American Type Culture Collection (VA, USA). GES-1 and GC cell lines were seeded into the Roswell Park Memorial Institute 1640 medium containing 10% fetal bovine serum (FBS) and 1% penicillin-streptomycin (P/S) and incubated. The cells were trypsinized and passaged until the cell confluence reached 90%. The LINC00467, miR-27b-3p, and STAT3 expression were evaluated by Reverse transcription quantitative polymerase chain reaction (RT-qPCR) and Western blot assay. Cell lines with the largest and least difference in relative expression from GES-1 were screened for follow-up assays.

### Cell grouping

BGC-823 cells were classified into seven groups and respectively subjected to transfection with short hairpin RNA (sh)-LINC00467, sh-negative control (NC), miR-27b-3p mimic, mimic NC, sh-LINC00467 + miR-27b-3p inhibitor, or sh-LINC00467 + inhibitor NC.

AGS cells were classified into seven groups as well and transfected with pcDNA-LINC00467, pcDNA-LINC00467 NC, miR-27b-3p inhibitor, inhibitor NC, pcDNA-LINC00467 + miR-27b-3p mimic, or pcDNA-LINC00467 + mimic NC.

These nucleotide sequences were purchased from GenePharma Co., Ltd. (Shanghai, China). After seeding onto 12-well plates for 24 h, cells were transfected. Lipofectamine 2000 (Invitrogen Inc., CA, USA) was utilized to mediate the transient transfection of the sequences into BGC-823 and AGS cells. Six-hour later, the medium was replaced. Cells were obtained 48 h after culture.

### RT-qPCR

The RNA extraction kits (Promega Corporation, WI, USA) were adopted for total RNA extraction. An ultraviolet spectrophotometer (Thermo Fisher Scientific Inc., MA, USA) was employed to measure RNA concentration and purity. RNAs (mRNA and lncRNA) were reversely transcribed into cDNA by GoldScript one-step RT-PCR Kit (Applied Biosystems, Inc., CA, USA); and reverse transcription for miRNA was conducted with Hairpin-it^TM^ miRNA quantitative detection kits (GenePharma). LINC00467, miR-27b-3p, and STAT3 levels were detected using an ABI 7900 fast real-time PCR system (Applied Biosystems, Carlsbad, CA, USA). U6 and glyceraldehyde phosphate dehydrogenase (GAPDH) were set as endogenous references of miR-27b-3p, LINC00467, and STAT3. The PCR primers (Supplementary Table 1) were designed by Shanghai Sangon Biotechnology Co., Ltd. (Shanghai, China). The 2^−△△Ct^ method was adopted for data analyzing.

### Western blot analysis

Proteins were performed with 10% sodium dodecyl sulfate-polyacrylamide gel electrophoresis and transferred onto membranes, which were sealed with 5% skim milk powder at 4 °C overnight. Subsequently, the membranes were incubated with primary antibody STAT3 (1: 1000, Abcam Inc., MA, USA) at 4 °C overnight, following 2-h incubation with the secondary antibody. The enhanced chemiluminescent reagent was used for development. Images were captured by Bio-rad chemiluminescent imaging system and matched software. GAPDH was set as the endogenous reference.

### 3- (4, 5-dimethylthiazol-2-yl) -5 (3-carboxymethoxyphenyl) -2- (4-sulfopheny) -2H-tet-razolium, inner salt (MTS) colorimetry

Transfected cells were seeded and incubated (3 duplicates were set in each group). A 10-min water bath at 37 °C was used to dissolve CellTiter 96^®^ AQueous One Solution Reagent (Promega). Every 100 μL medium in each well was supplemented with 20 μL CellTiter 96^®^ Aqueous One Solution Reagent at the 24th, 48th, and 72nd h of the transfection. The incubation lasted for 4 h and the absorbance at 490 nm was analyzed. Mean values of absorbance each day were used to graph the cell growth curves.

### Colony formation assay

Transfected GC cells were seeded into 24-well plates (100 cells/well). The cells were subjected to a 21-d incubation in a cell incubator_._ After medium removing, the cells were treated with 30-min fixation with 4% paraformaldehyde and 10-min staining with 0. 1% crystal violet. After staining, the wells were inverted and a transparent film with a grid was superimposed. Colonies were calculated and photographed under a microscope.

### Scratch test

Cells were seeded and cultured for 24 h. A scratch on the cell layers was caused by a sterile 200 μL pipette tip along the lines on the back of plates, then the nonadherent cells were removed. There were clear intervals after scratching, and the medium was replaced for continuous culture. The width of the scratch was observed and measured under a microscope at 0 h and 48 h, and the scratch healing rate was calculated.

### Transwell assay

Transwell chambers (pore diameter of 8 μm, Millipore Inc., MA, USA) coated with Matrigel were placed into 24-well plates and incubated for 10 min. The chambers were added with 300 μL serum-free RPMI-1640 medium and placed for 30 min. After 12-h starvation, cells were detached, centrifuged for 5 min, rinsed by serum-free medium and resuspended. The cell suspension concentration was adjusted into 2 × 10^4^ cells/mL, and 200 μL suspension was appended into the apical chambers. The bottom chambers were added with the 700 μL medium containing 10% FBS, following 48-h incubation. After medium removing, cells were subjected to fixation and staining with 0.5% crystal violet dye solution. Cells on the membrane were wiped off. Five visual fields were randomly selected. Cells were counted under a light microscope. The mean value was calculated.

### Flow cytometry

Apoptosis was evaluated by flow cytometry as previous record [22]. A flow cytometer (BD Biosciences, NJ, USA) was employed to determine apoptosis.

### Subcutaneous tumorigenesis in nude mice

An amount of 84 BALB/c nude mice (age: 4 w; weight: 20–22 g) were fed in animal rooms with steady temperature and humidity, and normal food and water. The nude mice were randomly classified into 14 groups (*n* = 6). BGC-823 cells: the blank, sh-LINC00467, sh-NC, miR-27b-3p mimic, mimic NC, sh-LINC00467 + miR-27b-3p inhibitor and sh-LINC00467 + inhibitor NC groups; AGS cells: the blank, pcDNA-LINC00467, pcDNA-LINC00467 NC, miR-27b-3p inhibitor, inhibitor NC, pcDNA-LINC00467 + miR-27b-3p mimic and pcDNA-LINC00467 + mimic NC groups.

Cells were amplified, collected, counted, and diluted into 1 × 10^7^ cells/mL cell suspension by phosphate-buffered saline (PBS). The nude mice were subjected to subcutaneous injection of 200 μL cell suspension at the right lateral axilla according to the grouping. When the xenografts could be observed by eyes, the length-diameter (L) and width-diameter (W) of the xenografts were measured every 4 d. According to the formula tumor volume (*V*) = (*L* × *W*^2^)/2, growth curves of the xenografts were graphed. Subsequently, the xenografts were harvested on a clean working table and An electronic balance was adopted for tumor weight measurement.

### Dual-luciferase reporter gene assay

Binding sites of LINC00467 and miR-27b-3p (or miR-27b-3p and STAT3) were predicted and analyzed by a bioinformatic website RNA22 (https://cm.jefferson.edu/rna22/Precomputed/). LINC00467 (or STAT3) wild type (WT) plasmid was constructed and the binding sites were mutated to establish the LINC00467 (or STAT3) mutant type (MUT) plasmid. The sequenced plasmids were transfected into BGC-823 and AGS cells respectively with mimic NC and miR-27b-3p mimic for 48 h. The luciferase detection kits (BioVision, CA, USA) and Glomax20/20 luminometer (Promega) were employed to assess the luciferase activity.

### RNA pull-down assay

Biotin-labeled miR-27b-3p WT and mutant MUT plasmid (50 nM each) were subjected to transfection with BGC-823 and AGS cells, respectively. After 48-h transfection, cells were subjected to 10-min incubation with specific cell lysate (Ambion, Austin, USA), Then, 50 mL sample cell lysate was subpackaged. The residual lysate was incubated with M-280 streptavidin magnetic beads (Sigma, MO, USA) pre-coated with RNase-free and yeast tRNA (Sigma) at 4 °C for 3 h, then washed twice washing with cold lysate, 3 times with low salt buffer, and once with high salt buffer. The bound RNA were purified using TRIzol reagent (Invitrogen) for further qRT-PCR analysis.

### Statistical analysis

The SPSS 21.0 software (IBM Corp. Armonk, NY, USA) was adopted for data analyzing. The measurement data were reported as mean ± standard deviation. The t test was adopted to compare data in two groups; one-way analysis of variance (ANOVA) and Tukey’s post hoc test were applied to compare multiple data in two more groups. The relationship between LINC00467 levels and clinicopathological features of GC was assessed by Fisher’s exact test or Chi-square test. The Kaplan–Meier was employed to analyze the survival prognosis of GC patients; and the interaction between LINC00467, miR-27b-3p, and STAT3 levels was examined by the Pearson test. *P*-value < 0.05 implied significant difference.

## Supplementary information

Supplementary Table 1-2

aj-checklist

cddiscovery-author-contribution-form

## Data Availability

The original contributions presented in the study are included in the article/Supplementary Material, further inquiries can be directed to the corresponding author.
