# Supplementary material for: Long intergenic non-protein coding RNA 467 inhibition elevates microRNA-27b-3p to repress malignant behaviors of gastric cancer cells via reducing STAT3
Source: Cell Death Discov. 2022 Mar 5;8:100. doi: 10.1038/s41420-022-00875-z (PMC8898310; doi:10.1038/s41420-022-00875-z)
Supplement: Supplementary file 1 — Supplementary Table 1-2 [file 41420_2022_875_MOESM1_ESM.docx]

**Supplementary Table 1** Primer sequences for the method of RT-qPCR

| Gene | Primer sequence (5’→3’) |
| --- | --- |
| miR-27b-3p | F: TTCACAGTGGCTAAGTTCTGC |
|  | R: Universal primer |
| U6 | F: GCTTCGGCAGCACATATACTAA |
|  | R: AACGCTTCACGAATTTGCGT |
| LINC00467 | F: GAGGTTGTCAAGTCGGACCA |
|  | R: GCCCTGGCATCTTCAGGATT |
| STAT3 | F: CTGGCCTTTGGTGTTGAAAT |
|  | R: AGGGCGAGGACCATAGAGG |
| GAPDH | F: TCAACGACCACTTTGTCAAGCTCA |
|  | R: GCTGGTGGTCCAGGGGTCTTACT |

Notes: F, forward; R, reverse; miR-27b-3p, microRNA-27b-3p; LINC00467, long intergenic non-protein coding RNA 00467; STAT3, signal transducer and activator of transcription 3; GAPDH, glyceraldehyde phosphate dehydrogenase.

**Supplementary Table 2** The correlation between the relative expression levels of LINC00467 and the clinicopathological characteristics of GC patients

| Clinicopathological features | n | LINC00467 | | *P* |
| --- | --- | --- | --- | --- |
|  | 52 | Low expression group (n = 26) | High expression group (n = 26) |  |
| Age |  |  |  | 0.404 |
| ≤58 years | 28 | 16 | 12 |  |
| >58 years | 24 | 10 | 14 |  |
| Gender |  |  |  | 0.572 |
| Male | 31 | 17 | 14 |  |
| Female | 21 | 9 | 12 |  |
| Site of tumor |  |  |  | 0.520 |
| Distal third | 11 | 6 | 5 |  |
| Middle third | 15 | 9 | 6 |  |
| Proximal stomach | 26 | 11 | 15 |  |
| Differentiation |  |  |  | 0.025 |
| High/ moderate | 25 | 17 | 8 |  |
| Poor | 27 | 9 | 18 |  |
| TNM stage |  |  |  | <0.001 |
| I–II | 26 | 21 | 5 |  |
| III–IV | 26 | 5 | 21 |  |
| Lymph node Metastasis |  |  |  | 0.024 |
| Yes | 29 | 10 | 19 |  |
| No | 23 | 16 | 7 |  |

Note: TNM, tumor node metastasis.
